# Efficient wavelength conversion of exchange magnons below 100 nm by magnetic coplanar waveguides

**DOI:** 10.1038/s41467-020-15265-1

**Published:** 2020-03-19

**Authors:** Ping Che, Korbinian Baumgaertl, Anna Kúkol’ová, Carsten Dubs, Dirk Grundler

**Affiliations:** 1 Laboratory of Nanoscale Magnetic Materials and Magnonics, Institute of Materials (IMX), École Polytechnique Fédérale de Lausanne (EPFL), 1015 Lausanne, Switzerland; 2 Laboratory of Semiconductor Materials, Institute of Materials (IMX), École Polytechnique Fédérale de Lausanne (EPFL), 1015 Lausanne, Switzerland; 3 INNOVENT e.V., Technologieentwicklung, Prüssingstr. 27B, 07745 Jena, Germany; 4 Institute of Microengineering (IMT), École Polytechnique Fédérale de Lausanne (EPFL), 1015 Lausanne, Switzerland

**Keywords:** Ferromagnetism, Magnetic properties and materials, Spintronics, Electronic and spintronic devices

## Abstract

Exchange magnons are essential for unprecedented miniaturization of GHz electronics and magnon-based logic. However, their efficient excitation via microwave fields is still a challenge. Current methods including nanocontacts and grating couplers require advanced nanofabrication tools which limit the broad usage. Here, we report efficient emission and detection of exchange magnons using micron-sized coplanar waveguides (CPWs) into which we integrated ferromagnetic (m) layers. We excited magnons in a broad frequency band with wavelengths *λ* down to 100 nm propagating over macroscopic distances in thin yttrium iron garnet. Applying time- and spatially resolved Brillouin light scattering as well as micromagnetic simulations we evidence a significant wavelength conversion process near mCPWs via tunable inhomogeneous fields. We show how optimized mCPWs can form microwave-to-magnon transducers providing phase-coherent exchange magnons with *λ* of 37 nm. Without any nanofabrication they allow one to harvest the advantages of nanomagnonics by antenna designs exploited in conventional microwave circuits.

## Abstract (web summary)

Magnons - collective excitations of electron spins - promise compact and fast electronics. However, the generation of short wave magnons is still quite challenging. Here, the authors demonstrate that by introducing a ferromagnetic layer, conventional coplanar waveguides can be used to efficiently generate such magnons.

## Introduction

Collective spin excitations have attracted growing attention in view of low-power-consuming information technologies that process the data without moving charges^1,2^. Here, exchange magnons with short wavelengths *λ* and correspondingly large wavevectors *k* = 2*π*∕*λ* are of special interest in the applied sciences when aiming at miniaturization of magnon-based logic and memory devices^3,4^. However, most of the experimental work on magnon-based computing and signal transmission has been conducted in the long-wavelength regime, where the spin waves are dominated by dipolar interaction^5–10^. In the technologically relevant frequency regime from a few to tens of GHz the on-chip excitation and detection of short-wave magnons are still challenging. The lack of efficient transducers is also detrimental in the fundamental sciences when experimentally exploring magnon scattering and band structures in noncollinear spin textures, such as skyrmion lattices^11,12^. Coplanar waveguides (CPWs) with lateral feature sizes down to 125 nm have not yet been sufficient for efficient emission of short-wave magnons^13–15^. Instead parametric pumping in the nonlinear regime has been utilized to obtain exchange magnons with *k* > 20 rad μm^−1^^16–18^. Concerning emission of such magnons in the linear regime, nonuniform spin textures^19–22^, magnetic interfaces^23,24^, and the magnonic grating coupler effect^25–27^ have been explored. Magnons with *λ* ≈ 50 nm (*k* ≈ 120 rad μm^−1^) were induced in thin yttrium iron garnet (YIG) by means of a grating coupler consisting of a periodic lattice of parallel nanostripes^28^. Here, the nanostripes were prepared by nanolithography, and the gap width *g* between them amounted to 100 nm. Such challenging nanofabrication impedes broad application and is an obstacle to progress in this field. The conversion factor *η* = *g*∕*λ* between critical lateral dimension *g* and wavelength amounted to 2. Larger factors *η* and the excitation of exchange magnons without nanofabrication are of high relevance for advancing magnonics both as a research field and possible information technology. A nonuniform effective field in a ferromagnetic film was suggested to reduce magnon wavelengths via a conversion process^29–32^. However, the experimental observation of exchange magnons with *k* > 20 rad μm^−1^ has not yet been reported.

**Fig. 1:**
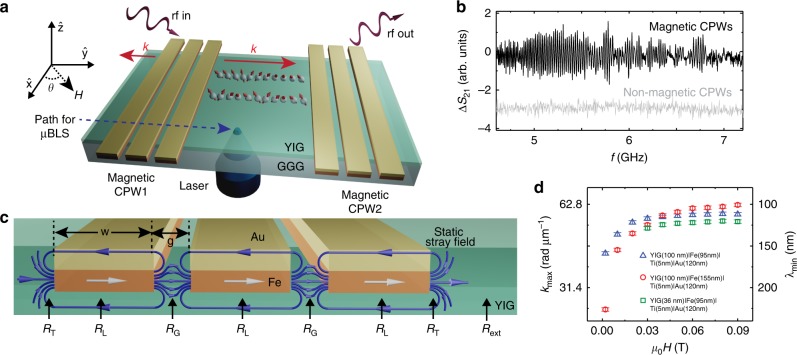
Schematic diagram and high-frequency modes emitted by mCPWs. **a** Sketch of the experiment showing mCPWs on YIG and microfocus BLS performed through the GGG substrate. The red arrows indicate propagation directions of magnons. The blue arrow indicates the path along which local BLS spectra were obtained. **b** Δ*S*_21_ reflecting propagating magnons (oscillatory signal) detected electrically at 0.09 T (*θ* = 88°) with mCPWs (black) and the signal obtained by nonmagnetic conventional CPWs (gray). **c** Schematic diagram of the stray field generated by Fe layers around an mCPW. White arrows display the magnetization component along the field applied at *θ* = 90°. Blue lines with arrows illustrate the stray field of Fe. We define regions (R) underneath ground and signal lines (L), in gaps (G), and a transition (T) region between the mCPW and the bare YIG which is denoted by region R_ext_ further away from the mCPW. **d** Maximum wavevectors ${k}_{\max }$ (and corresponding minimum wavelengths ${\lambda }_{\min }$) extracted from Δ*S*_21_ data taken on three samples (symbols are labeled in the inset) by considering the relevant dispersion relations in bare YIG given by Kalinikos and Slavin^38^. The error bar refers to the frequency resolution of the VNA. For each sample, the field was reduced from *μ*_0_*H* = 0.09 T to 0.002 T. Source data are provided as a Source Data file.

In this work, we introduce magnetic coplanar waveguides (mCPWs) consisting of Fe∣Ti∣Au with micrometer-scale lateral dimensions, and report excitation and detection of exchange magnons (Fig. 1a, b). Using not yet optimized mCPWs we extracted *k* = 62.4 rad μm^−1^ (*λ* = 100 ± 2 nm) for emitted magnons at 7.02 GHz. The effective field in YIG underneath the mCPWs was modified by the stray field of the polycrystalline Fe layer (Fig. 1c), and induced wavelength conversion giving rise to on-chip excitation and detection of exchange-dominated magnons (Fig. 1d). Avoiding challenging electron beam lithography on insulating YIG and utilizing photolithography for CPWs with a gap width *g* of 1.4 μm we realized a large conversion factor of *η* = 14. This value goes beyond previously reported methods based on, e.g., the magnonic grating coupler^25^ or a nanocontact^33^. Preparing the mCPWs on YIG, we evidence emission, detection, and the transport of phase-coherent exchange magnons over macroscopic distances via both electrical and optical methods. We outline how to optimize mCPWs and obtain *k* ≈170 rad μm^−1^.

## Results

### Broadband magnon excitation by mCPWs

**Fig. 2:**
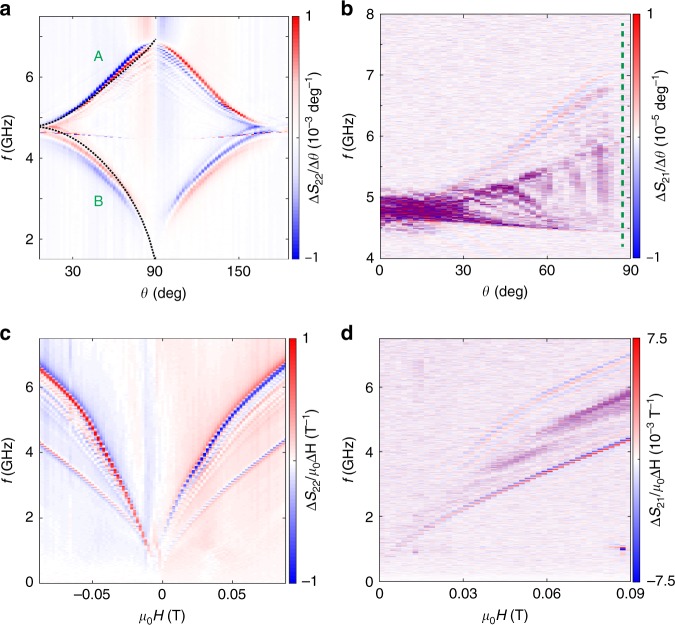
Spectra of magnons emitted by mCPWs for fields applied under different angles *θ*. **a** Reflection and **b** transmission spectra taken for a field of 0.09 T, where *θ* was varied in a step-wise manner. The data were obtained on thin YIG with mCPWs consisting of Fe(155 nm)∣Ti(5 nm)∣Au(120 nm) (sample 1). Black dotted lines in **a** are resonance frequencies calculated by considering simulated effective fields *B*_eff_ in Kittel's formula^35^. They reflect *B*_eff_ taken at the central positions in regions R_G_ (top curve A) and R_L_ (bottom curve B). The dashed vertical line in **b** indicates the angle at which the top line spectrum of Fig. 1b is extracted. **c** Reflection and **d** transmission spectra of sample 1 as a function of field applied at *θ* = 90°. The color scales are defined in the legends. The contrast was optimized to highlight the broadband magnon excitation. For data sets in **c** and **d**, the field was reduced from 0.09 T to  −0.09 T.

Two parallel mCPWs consisting of a layer sequence Fe∣Ti∣Au were integrated on a YIG thin film as illustrated in Fig. 1a. Their center-to-center separation *s*_cc_ was 35  μm. The width of signal and ground lines was *w* = 2.1 μm with a gap of width *g* = 1.4 μm in between. Their lengths amounted to 125 μm. Seven devices with different thicknesses of the Fe layer of the mCPWs were prepared on either 100 nm or 36 nm thick YIG^34^ (Supplementary Table I and Supplementary Fig. 1). Radio-frequency currents were injected into the coplanar waveguides and induced an inhomogeneous microwave magnetic field **h**_rf_. The Fourier analysis of *h*_rf_ provided a set of wavevectors **k**_*i*_ with *i* = 1, 2, 3, . . .  (Supplementary Fig. 2). Scattering parameters *S*_*xy*_ with *x*, *y* = 1, 2 were measured by a vector network analyzer (VNA). Both, magnitude and phase information were collected. The reflection coefficients *S*_11_ and *S*_22_ corresponded to spectra displaying magnons excited near mCPW1 and mCPW2, respectively. The transmission coefficients *S*_21_ and *S*_12_ detected magnons propagating through the bare YIG film between mCPW1 and mCPW2. An in-plane field *H* was applied under different angles *θ* (Fig. 1a). Magnitude data Δ*S*_21_ of sample 1 with mCPWs consisting of Fe(155 nm)∣Ti(5 nm)∣Au(120 nm) and sample 4 with nonmagnetic CPWs consisting of Fe(0 nm)∣Ti(5 nm)∣Au(120 nm) (Supplementary Table I) are compared in Fig. 1b for *μ*_0_*H* = 0.09 T applied at *θ* = 88°. The spectrum of sample 1 contains prominent oscillations, indicating propagating magnons in YIG between mCPWs. The propagating magnons cover a frequency band of ~2.5 GHz. The spectrum of sample 4 taking under the same applied field condition shows the noisy background signal, but no propagating magnon signal. Figure 2a (b) displays the angular dependence of reflection (transmission) signals when a field of 0.09 T is rotated by *θ* with respect to CPWs. It is found that the main magnon mode observed in sample 1 at *θ* = 0° splits into two prominent branches A and B when the angle *θ* is increased from 0° to 90°. The modes merge again when *θ* is further increased and reaches 180°. The large splitting into two prominent branches with increasing *θ* is not found in case of nonmagnetic CPWs used on sample 4 (Supplementary Fig. 2). A detailed inspection of spectra at *θ* = 88° (see green broken line in Fig. 2b) reveals magnon excitation over a broad frequency regime as evidenced by the oscillating signal in the upper trace of Fig. 1b. When the angle *θ* is fixed at 90° and the field strength is varied from 0.09 T to −0.09 T (Fig. 2c, d), the multifrequency excitation of magnons over a frequency band of more than 2.5 GHz at high *H* is clearly resolved. Before discussing the relevant wavevectors *k* for magnons detected in transmission spectra such as those displayed in Figs. 1b, 2b, d, it is instructive to analyze the stray fields generated by mCPWs as sketched in Fig. 1c.

### Local effective fields in YIG

**Fig. 3:**
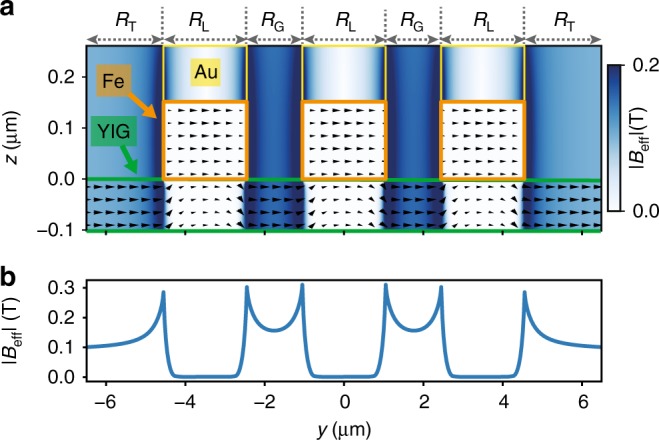
Micromagnetic simulation and line plot of effective magnetic field generated by mCPW. **a** Cross-sectional view of an mCPW on YIG where the different layers of Au, Fe, and YIG are indicated in yellow, orange, and green color, respectively. Arrows are extracted from micromagnetic simulations, and display the local magnetization vectors in the *y*, z plane for a field of 0.09 T applied in positive *y*-direction (*θ* = 90°). The color coding of the background (legend on the right) indicates the magnetic field *B*_eff_ taken from simulations, which varies locally and enters the equation of motion. **b** Line plot of the absolute *B*_eff_ inside YIG along the path of BLS spectroscopy as indicated in Fig. 1a. Source data are provided as a Source Data file.

To explore the origin of branches A and B found in Fig. 2a for *θ* ≠ 0°, we studied the static magnetization of an mCPW with dimensions of sample 1 using micromagnetic simulations (“Methods”). We display the considered layer sequence in Fig. 3a and the simulated effective magnetic field **B**_eff_ in Fig. 3b assuming an external magnetic field of 0.09 T applied at *θ* = 90° to the mCPW. The magnitude of **B**_eff_ is evaluated within the thin YIG film. *B*_eff_ shows a characteristic variation underneath and next to the mCPW. In the following section, we discuss the simulated field strength in four distinct regions (R) inside YIG. The central region underneath the signal and ground lines (L) of the mCPW is labeled by R_L_. Here we find the lowest field value, because the applied field and stray field of Fe are of opposing direction and cancel each other almost perfectly. In the gaps (G), i.e., the region between signal and ground lines indicated by R_G_, we find high-field values as externally applied and stray fields exhibit the same direction and sum up. Further away from the mCPW at large coordinates *y*, we obtain a field strength consistent with the externally (ext) applied field. Here, we label the region by R_ext_ (Fig. 1c). For the discussion, it is instructive to consider the field strength also in the transition (T) region labeled by R_T_, in which the effective field is found to be larger than the external field and the microwave field *h*_rf_ is strong producing a large torque. Even larger inhomogeneous fields can be found in the gaps (G) enclosed by two Fe strips. Also in R_G_ the out-of-plane microwave component is strong for resonant excitation of spin waves. When introducing such different effective fields *B*_eff_ into the equation of motion of spin precession^35^, different eigenfrequencies are expected. They roughly scale with *B*_eff_. Considering the equation of motion, we tentatively attribute the branch A which we observe at high frequency in the reflection signal of Fig. 2a to prominent absorption in (gap) regions R_G_. Branch B is then attributed to absorption in regions R_L_ underneath the CPW lines. To substantiate the allocation, we performed micromagnetic simulations for 0.09 T applied under different angles *θ* with respect to the mCPW and extracted angle-dependent *B*_eff_(*y*) quantitatively. Introducing *B*_eff_(*y*) extracted at central positions of R_G_ and R_L_ into Kittel’s formula^35^ for thin YIG, we obtained the two dotted black lines displayed in Fig. 2a. They model the angular dependencies of the two prominent branches A and B well. The agreement indicates that the inhomogeneous effective fields induced in YIG by the stray fields of the Fe stripes are key to understand the magnon modes excited and detected by mCPWs.

### Spatially resolved magnon spectra

**Fig. 4:**
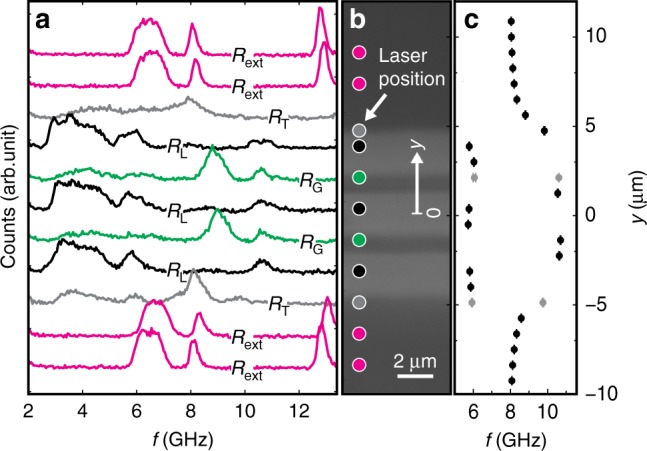
Spatially resolved magnon spectra at the mCPWs. **a** Spectra of thermally excited magnons in sample 1 measured by  *μ*BLS for *μ*_0_*H* = 0.15 T and *θ* = 90° at different laser-spot positions indicated by circles in the **b** optical image taken by the BLS microscope. The systematic variation of spectra depending on laser-spot positions is color-coded consistently in **a** and **b**. **c** Variation of the frequency attributed to the PSSW1 mode along the path of BLS spectroscopy (black and gray symbols). The gray symbols indicate spectra taken at the boundary between regions R_G_ and R_L_. In this case, the spectra contained two PSSW1 peaks which were consistent with YIG subjected to either high or low effective field. We attribute this observation to the finite diameter of the BLS laser beam probing two regions at the same time. Peak positions were extracted by locally fitting the magnon spectra with a Gaussian function, error bars indicate the standard deviation of the fit. Source data are provided as a Source Data file.

To spatially resolve effective field-induced frequency variations close to an mCPW, we measured thermally excited magnon spectra at characteristic positions beneath and close to mCPW1 of sample 1 via microfocus Brillouin light scattering (*μ*BLS)^36,37^. A laser with wavelength of 473 nm was focused from the backside of sample 1 through the transparent gadolinium gallium garnet (GGG) substrate to a diffraction-limited spot on the YIG surface (Fig. 1a). This allowed for the investigation of the different regions defined in Fig. 3a (top axis). To better detect all the different modes, we applied a field of *μ*_0_*H* = 0.15 T at *θ* = 90° and thereby shifted eigenfrequencies to frequency values for which the interferometer provided the largest signal-to-noise ratio. Spectra marked with R_ext_ in Fig. 4 were recorded sufficiently away from the mCPW to reflect excitations in bare YIG. Here, considering the measured frequency range from 2 GHz to 13.5 GHz, three distinct peaks were observed. By comparison with an analytical dispersion relation for 100 nm thick YIG^38^, we attributed the broad main peak between about 6 and 7 GHz to the continuum of magnons exhibiting in-plane wavevectors ∣*k*∣ from zero up to  ~20 rad μm^−1^, which are allowed by the focussing optics (Methods)^16^. The mode at 8.1 GHz (12.8 GHz) is identified as first (second) perpendicular standing spin wave PSSW1 (PSSW2). The PSSW1 peak exhibits a small linewidth. When the laser-spot approaches mCPW1, the magnon peaks shift to higher and higher frequencies. In Fig. 4c, we track the position-dependent frequency of PSSW1. From 8.1 GHz at R_ext_, the PSSW1 frequency increases to around 10.6 GHz at R_G_ in the gap between the magnetic stripes (spectra are found to be symmetric to the mirror plane of the CPW). In regions R_L_ underneath the CPW lines incorporating Fe stripes, the PSSW1 frequency is significantly reduced and exhibits 5.8 GHz. Qualitatively, the frequency variation reflects the spatial variation of the effective field displayed in Fig. 3b. We attribute the observed shifts of PSSW1 hence to a varying effective field in YIG caused by the stray field of Fe. The frequency variation from 8.1 to 10.6 GHz is consistent with a variation of *B*_eff_ by 88 mT following the formalism of ref. ^38^. This value agrees quantitatively well with the simulated field difference between the central position in R_G_ and R_ext_.

We note that also peak shapes are modified in the vicinity of the mCPW. In R_L_, the observed fundamental mode is broader than in R_ext_. We speculate that the broadening originates from the finite diameter of the probing laser spot which detects magnons subjected to different effective fields. At R_G_, the width of the main peak is slightly narrower than at R_ext_. We attribute this observation to a reduced range of allowed wavevectors and speculate that magnons are partially confined in the inhomogeneous field between signal and ground lines. As a consequence, long magnon wavelengths in *y*-direction are not allowed. Measurements conducted at *μ*_0_*H* = 0.087 T (Supplementary Fig. 3) showed similar characteristics. Only low-frequency modes were not fully resolved due to the restricted frequency range of the interferometer stated above.

### Wavevectors of transmitted magnons

The oscillating signal Δ*S*_21_ shown in Fig. 1b originated from magnons that were phase-coherently detected in transmission configuration and propagated through the bare YIG film between mCPW1 and mCPW2 at specific frequencies *f*. The edge-to-edge separation Δ*y*_e-e_ of CPWs was 25.9 μm. We calculated the dispersion relations *f*(*k*) for YIG films of three different thicknesses according to the formalism provided by Kalinikos and Slavin^38^ (“Methods”). For these calculations, we considered the field *H* applied in the VNA spectroscopy setup. Our simulations showed that already about 2 μm away from mCPWs, the effective field in YIG reduced to almost the applied field. From the calculated *f*(*k*), we extracted the wavevectors *k* which were relevant for the magnons propagating through YIG at different frequencies *f*. In Fig. 1d, we display the maximum wavevectors ${k}_{\max }$ (minimum wavelengths ${\lambda }_{\min }$) found in three differently thick YIG films with mCPWs. In all cases, the field *H* was applied at *θ* = 90°, i.e., the magnetization **M** was parallel to **k** and magnons propagated in backward volume magnetostatic wave configuration through YIG. For all samples, the maximum wavevector of propagating magnons increased with increasing *H*. Such an increase was not observed for nonmagnetic CPWs. Remarkably for a given field, mCPWs excite wavevectors smaller than ${k}_{\max }$ as well. The oscillating signal of propagating magnons observed in Fig. 1b (upper trace) reflects wavevectors varying quasi-continuously by about one order of magnitude. This large tunability of *k* is not known from the grating coupler effect.

### Propagation of exchange magnons

**Fig. 5:**
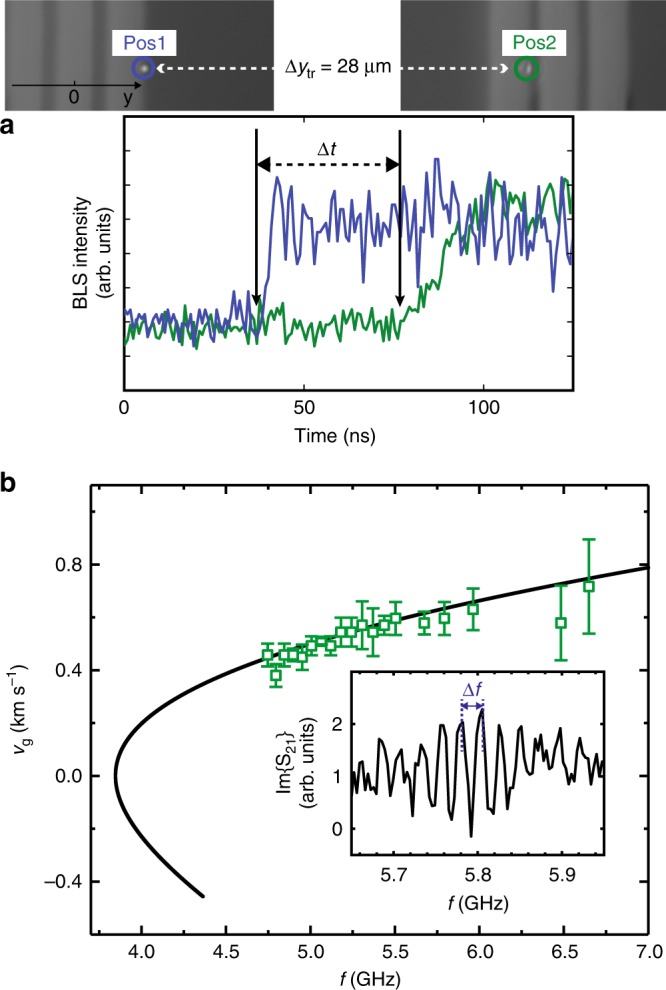
Group velocities of exchange magnons. **a** Emission of exchange magnons evidenced by BLS. Time-resolved magnon intensities measured at indicated positions Pos1 of mCPW1 and Pos2 of mCPW2 are depicted as blue and green line, respectively. The measurement was conducted at *μ*_0_*H* = 0.087 T. A microwave pulse with 6.64 GHz was applied to mCPW1. A rise in BLS intensity was first observed at Pos1 and with a delay of Δ*t* = 43 ± 3 ns at Pos2, which was separated from Pos1 by $\Delta {y}_{{\rm{tr}}}$ = 28 μm. **b** Average group velocities (open squares) extracted for sample 1 from VNA transmission signals using *v*_g_ = Δ*f*  × Δ*y*_e-e_. The solid curve represents calculated velocities *v*_g_ assuming a backward volume spin-wave dispersion relation at 0.09 T (see “Methods”). Inset: Δ*f* was extracted from oscillations in the imaginary (Im) part of the transmitted magnon spectra *S*_21_, when at least three periods of oscillations appeared. Error bars are the standard deviation of *v*_g_ indicated by those three oscillations. Source data are provided as a Source Data file.

We measured the velocity of propagating magnons emitted by an mCPW via time-resolved  *μ*BLS. A microwave signal generator equipped with a microwave switch was connected to mCPW1 of sample 1 and applied a microwave signal at *f* = 6.64 GHz. A field of *μ*_0_*H* = (0.087 ± 0.002) T was applied at *θ* = 90^∘^ to the sample. Once the microwave switch was opened (rise time of ≤4 ns), an increase of the BLS counts was detected at CPW1 in region R_T_ as shown in Fig. 5a. At R_G_ of CPW2, the signal was detected with a time delay Δ*t* = 43 ± 3 ns (defined at 10% increase of the rising edge). The distance between the two measured spots amounted to $\Delta {y}_{{\rm{tr}}}=28\ \upmu {\rm{m}}$. Considering $\overline{{v}_{{\rm{g}}}}=\Delta {y}_{{\rm{tr}}}/\Delta t$, we calculated an average velocity $\overline{{v}_{{\rm{g}}}}$ of (0.65 ± 0.05) km s^−1^. This value compared well with the group velocity *v*_g_ of a short-wave magnon calculated for 6.64 GHz (compare solid line in Fig. 5b). In bare YIG, the frequency of 6.64 GHz corresponded to a magnon with a wavelength of 105(±1) nm according to the Kalinkos and Slavin formalism^38^. The symbols displayed in Fig. 5b refer to velocities extracted from oscillation periods Δ*f* occurring in VNA data *S*_21_ as the one shown in the inset. Here, sample 1 with 155 nm thick Fe at 0.09 T was evaluated. We calculated $\overline{{v}_{{\rm{g}}}}$ from $\overline{{v}_{{\rm{g}}}}=\Delta f\times {s}_{{\rm{eff}}}$^39^, where the parameter *s*_eff_ estimated the length of YIG over which the propagating magnon accumulated the relevant phase shift Δ*φ* between mCPW1 and mCPW2. For the symbols shown in Fig. 5b, we assumed *s*_eff_ = *Δy*_e-e_ = 25.9 μm. The good agreement between extracted data (symbols) and the predicted group velocities indicates that the phase shift Δ*φ* along *s*_cc_ is accumulated mainly across region R_ext_ where we assume the propagating magnons to exhibit the maximum wavevector *k* for the given applied field and excitation frequency *f*. In Fig. 5b, velocities increase with frequency. This behavior is consistent with exchange-dominated magnons.

**Fig. 6:**
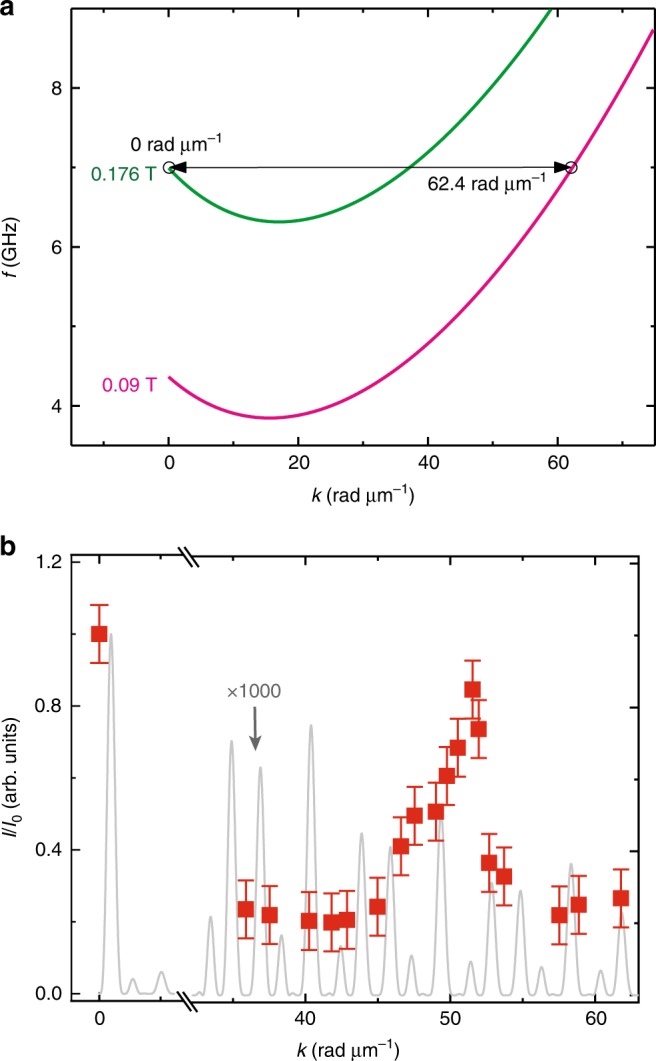
Wavelength conversion and magnon signal strengths. **a** Dispersion relations of magnons which are calculated using the formalism of Kalinikos and Slavin^38^ in the gap region R_G_ of an mCPW (green) and in the region R_ext_ of bare YIG (pink) considering sample 1. **b** Normalized signal strength *I*∕*I*_0_ of magnon resonances (squares) extracted from data such as those shown in Fig. 2d at 0.09 T. *I* (*I*_0_) is the peak-to-peak signal strength in ΔS_21_ at *k* (*k*_1_ of a CPW). Error bars represent the noise level observed in the spectra. The solid gray line reflects the Fourier transformation (power spectrum) of the in-plane radio-frequency field component of *h*_rf_ of a nonmagnetic CPW. Source data are provided as a Source Data file.

At *f* = 6.64 GHz, the magnon wavelength amounted to 105 nm. Such a short wavelength did not allow us to detect the magnon directly in the microfocus-BLS experiment on bare YIG, as its diffraction limit amounted to about 300 nm. Indeed, in the YIG film between the mCPWs we did not detect an increase in BLS counts when the microwave pulse was applied. Only in regions R_G_ and R_T_ representing large effective fields in YIG, we recorded increased BLS counts at the excitation frequency. Strikingly, the pulsed magnon signals were detected at both R_G_ and R_T_ regions of mCPW2. We attribute this to a significant conversion of wavelength for a magnon propagating from the low-field region of bare YIG into a region of large effective field created by the stray field of Fe. In our case, the wavelength was increased by a factor of at least three to become optically detectable. The conversion process is sketched in Fig. 6a: at fixed excitation frequency a magnon adjusts its wavevector *k* as given by the dispersion relation that is valid in the specific region of the sample. The conversion process was reported for dipolarly dominated spin waves^30,40,41^, but not yet for exchange magnons.

## Discussion

Our experiments based on broadband VNA spectroscopy, spatially and time-resolved Brillouin light scattering as well as micromagnetic simulations indicate that CPWs incorporating a ferromagnetic layer of Fe allow one to generate short-wave exchange magnons in thin YIG films. Close to the edge of the CPW, the stray field of Fe generates locally a large effective field. This field enters the equation of motion and leads locally to a large eigenfrequency of spin precession at relatively small *k* (large wavelength determined by either the geometry of the CPW or the localization length in the inhomogeneous *B*_eff_) (Fig. 6a). When propagating away from the mCPW, the magnon enters a region of small magnetic field in which the dispersion relation imposes a large *k* on the magnon. To obey energy conservation, the wavevector hence transforms along the propagation path and undergoes an up-conversion in *k* (Fig. 6a). In sample 1 for *f* = 7.02 GHz and *μ*_0_*H* = 0.09 T applied along *y*-direction, the wavevector is converted to ${k}_{\max }=62.4$ rad μm^−1^ when the magnon propagates from region R_G_ at the mCPW to position R_ext_ in bare YIG.

In Fig. 1d, we summarize the maximum wavevectors ${k}_{\max }$ attributed to the high-frequency magnons propagating through the bare YIG of three different thicknesses. These maximum wavevectors vary with the strength of *H* applied at *θ* = 90°. A reduction of the applied field leads to a decrease of ${k}_{\max }$ from 62.4 at 0.09 T to about 28 rad μm^−1^ at 0.002 T. We attribute the decrease to the variation of magnetization *M* in the Fe stripes. With decreasing *H* magnetic moments in Fe turn into a direction collinear with the long axis. Thereby the peak-to-peak variation of effective fields in YIG next to the mCPWs is less pronounced. We note that at *μ*_0_*H* = 0.002 T, the evaluated wavevector is still larger than expected from a nonmagnetic CPW. We attribute this observation to either the hysteresis of the Fe stripe (i.e., a finite remanent magnetization component of Fe along *θ* = 90°) or the differences in magnetic susceptibilities of Fe and YIG^24^. The observation of a significant wavelength-conversion process in very small applied field is promising in view of low-power consuming and compact magnonic applications, where an additional biasing magnetic field is avoided and instead electric fields control the magnonic functionality^42,43^.

The transmission signals *S*_21_ showed that both up- and downconversion of *k* took place. Via downconversion the spin-precessional motion of an incoming magnon induced a voltage in the metallic receiver CPW, which was phase-coherently detected by the VNA. In Fig. 6b, we display relative signal strengths *I*∕*I*_0_, depending on the wavevector *k* which we attributed to the magnons in bare YIG at 0.09 T. *I* is the signal strength in Δ*S*_21_ of the corresponding magnon, and *I*_0_ is the signal strength of the most prominent low-frequency branch *k*_1_. Strikingly *I*∕*I*_0_ of magnons with *k* ≈ 52  rad μm^−1^ is ~86% of the signal of magnons at *k*_1_. For a nonmagnetic CPW, one would expect *I*∕*I*_0_ of only ~0.04% (gray curve), i.e., more than three orders of magnitude smaller. The wavelength-conversion process hence offers a powerful methodology which does not require the nanolithography established for grating couplers or nanopillars. At the same time, mCPWs offer a broad frequency regime for magnon emission (Fig. 1b) not provided by grating couplers. Considering spatially resolved BLS data performed at 0.087 T (Supplementary Fig. 3), we suppose that the large signals *I*∕*I*_0_ for *k* near 52 rad μm^−1^ are attributed to spin waves excited (and detected) in R_T_ right at the outer edges of the mCPW. Here, both *B*_eff_(*y*) and the exciting microwave field *h*_rf_ exhibit large strengths. For propagation at wavevectors *k* > 52 rad μm^−1^, the gap regions R_G_ of mCPWs (Fig. 1b) provide the high effective fields that are required for resonant excitation in YIG. The corresponding spin waves undergo additional conversion processes when passing through the inhomogeneous *B*_eff_(*y*) induced by the ground lines. The reduction of *I*∕*I*_0_ observed for propagating magnons at large *k* is most likely caused by these processes inducing e.g., partial reflection. Still, the relative efficiencies for magnon emission and detection at large *k* are overall similar to the grating couplers^26,28^.

In Fig. 1d, we observe a reduction of ${k}_{\max }$ by ~10% when using an Fe thickness *δ* of 95 nm (sample 2) instead of 155 nm (sample 1). The observation is consistent with micromagnetic simulations predicting less peak-to-peak variation of *B*_eff_(*y*) inside YIG for decreasing *δ*. Correspondingly, the frequency splitting between branches A and B (Fig. 2a) depends on *δ* (Supplementary Fig. 1). The thickness *δ* hence offers control over the frequency band of magnon emission and detection. For a fixed Fe thickness of *δ* = 95 nm, we find ${k}_{\max }$ to decrease with decreasing thickness of YIG (compare sample 2 and sample 5 in Fig. 1d). The observed reduction in ${k}_{\max }$ reflects the change in magnon dispersion relation *f*(*k*), depending on the YIG thickness. Still the wavelength conversion is found to be significant for the small YIG thickness of 36 nm, and hence advantageous for coherent nanomagnonics^13^. To optimize mCPWs and further reduce *λ*, it is suggested to embed the ferromagnetic layers into YIG and thereby reduce the demagnetization effect in the Fe (Supplementary Fig. 4a, b). In this Schloemann-type configuration^29^, the effective field in YIG would be locally further increased enabling correspondingly higher excitation frequencies close to the CPW. In case of embedded FeCo with a saturation magnetization *M*_S_ = 1950 kA m^−1^^44^, our dynamic simulations suggest excited spin waves with *λ* = 37 nm at 28 GHz in YIG (Supplementary Fig. 4c–e), corresponding to *k* ≈170 rad μm^−1^. We argue that the wavelength conversion based on mCPWs is very versatile and works in both insulting and metallic magnets, in nanoscopic ferromagnetic waveguides^45^ and skyrmion-hosting materials^46^. The magnons with ${k}_{\max }=62.4$ rad μm^−1^ reported here possess wavelengths shorter than the minimum ones reported so far for nonmagnetic microwave waveguides^14,15,27^ and signals are about three orders of magnitude larger (Fig. 6b). To quantitatively understand the magnon amplitudes induced by mCPWs and model the frequency-dependent signal variation observed in Fig. 1b, a formalism needs to be developed, which, in the spin-precessional equation of motion, considers both the inhomogeneous effective field *B*_eff_(*y*) and noncollinear spin structures (Fig. 3a) as well as local differences in magnetic susceptibilities^24^.

In conclusion, we reported the emission and detection of short-wave magnons in YIG from CPWs incorporating ferromagnetic layers. Their stray field induced a nonuniform magnetic field. The non-uniformity was strong when the in-plane external magnetic field was applied perpendicular to the long axis of the mCPWs, i.e., perpendicular to the incorporated Fe stripes. The observed conversion efficiency was large, both concerning wavevector *k* and signal strength. Local wavelength conversion also allowed us to study propagating exchange magnons via optical detection. Importantly, for the fabrication only photolithography was needed and a conventional microwave antenna design was suitable. We find that mCPWs emit magnons over a broad frequency band, and thereby allow for continuous tuning of exchange magnons at telecommunication frequencies. This is different from grating couplers which emit at specific frequencies defined by discrete reciprocal lattice vectors. A growth-induced anisotropy axis for the ferromagnetic layer could stabilize a magnetization component transverse to the CPW in zero magnetic field, thereby enabling low-power consuming magnonic devices based on mCPWs. Wavelengths down to below 40 nm are achievable. We expect these features to make mCPWs a very versatile microwave-to-magnon transducer technology and promote advancements in experimental nanomagnonics both in the fundamental and applied sciences.

## Methods

### Sample fabrication

The mCPWs were fabricated on YIG thin films with thicknesses of either 36 nm or 100 nm by means of photolithography and lift-off processing. The single-crystalline YIG films were grown on (111) GGG substrates by liquid-phase epitaxy (LPE). The 36 nm thick YIG was grown at INNOVENT e.V. in Jena, Germany, on a 1-inch GGG wafer from a PbO-B_2_O_3_-based high-temperature solution using a standard dipping technique^34^. The 100 nm thick YIG was deposited on a 3-inch wafer via LPE, and purchased commercially from the company Matesy GmbH in Jena, Germany. Double layers of photoresist were coated on the YIG, and the CPW structures were written directly by a laser lithography system. Fe was first thermally evaporated in one chamber. Second, the chip was moved to another chamber for Ti and Au thermal evaporation. Seven devices were prepared. We explored three different thicknesses of Fe (155 nm, 95 nm, and 17 nm). The Au (Ti) layers were kept at a constant thickness of 120 nm (5 nm) (Supplementary Table I). The nonmagnetic reference CPW did not contain an Fe layer, and consisted of Au and Ti only.

### All electric broadband spin-wave spectroscopy

Magnons were electrically excited and detected on a broadband spectroscopy setup with a microwave probe station. Microwave currents *i*_rf_ from 10 MHz to 26.5 GHz were extracted from one port of a vector network analyzer (VNA), and injected into the coplanar waveguide using the high-frequency coaxial cable and probe. The dynamic microwave field **h**_rf_ generated by *i*_rf_ applied the dynamic torques on the magnetization and excited the magnons in the YIG thin film underneath the CPW. We report VNA experiments performed in the linear regime at −15 dBm, i.e., at a power of 0.03 mW. In the center of the 100 nm thick YIG beneath the signal line of a bare CPW without Fe, we estimated the strength of *μ*_0_**h**_*y*,rf_^47^ to be 0.68 mT at −15 dBm. The additional Fe layer of an mCPW increased the relevant distance in *z*-direction by 155 nm corresponding to *μ*_0_**h**_*y*,rf_ = 0.61 mT in the YIG center. The reduction in excitation strength amounts to ~10%. From −28 dBm to −5 dBm, we observed that the measured scattering parameter scaled linearly with the microwave signal. At −15 dBm, the main resonance in YIG was red-shifted by 46 MHz compared with −28 dBm. Considering the temperature-dependent resonance frequencies reported in ref. ^48^, we estimate a temperature increase of ~6 K. The wavevectors of the magnons were defined by the geometric design in conventional nonmagnetic CPWs as reported earlier, but were modified when mCPWs were used. In mCPW2, the microwave currents induced by the propagated magnons were probed via a coaxial cable by a port of the VNA, and corresponding scattering parameters were analyzed. The setup was calibrated using the standard kit designed for coaxial microwave probes of 50 Ohm impedance matching before measurements. External magnetic fields were applied in the *x*, *y*-plane as indicated in Fig.1a. The reflection and transmission spectra of angular-dependent measurements with mCPWs are plotted in Fig. 2a, b. The spectra were obtained while *μ*_0_*H* = 0.09 T was applied, and *θ* was varied from −2° to 200°. Field-dependent reflection measurements taken at *θ* = 90 are depicted in Fig. 2c. Figure 2d shows the transmission spectra for *μ*_0_*H* scanned from 0.09 T to 0 T. To enhance the signal-to-noise ratio and remove drifts in the background signal in Figs. 1b and 2, we display Δ*S* which reflects the difference in scattering parameters taken at successive field values or angles.

### Brillouin light scattering

Microfocus Brillouin light-scattering (*μ*BLS) measurements were conducted at room temperature. A monochromatic continuous-wave solid-state laser with a wavelength of 473 nm and power of  <1 mW was focused through the sample backside to a diffraction-limited-spot using a specially corrected ×100  objective lens with a large numerical aperture of *NA* = 0.85. The diffraction limit corresponded to a maximum accessible wavevector *k* of $4\pi \sin \phi /(473\ {\rm{nm}})=22.6$ rad  μm^−1^, considering $\sin \phi =NA=0.85$ in air (*ϕ* is the incident angle, *NA* is the numerical aperture). We tested the limit experimentally on propagating spin waves in YIG while focussing through the transparent GGG substrate. We detected propagating spin waves with wavelengths down to 330 nm corresponding to a wavevector of 19 rad  μm^−1^. Permanent magnets were used to apply external magnetic fields in the plane of the YIG either along or perpendicular to the CPWs. Backscattered light was analyzed with a six-pass Fabry–Perot interferometer TFP-2 (JRS Scientific Instruments). The recorded BLS signal is proportional to the square of the amplitude of the dynamic magnetization at the position of the laser spot. The sample was mounted on a closed-loop piezo-stage, and spatial maps were obtained by scanning the laser spot over the sample. mCPW1 was wire-bonded to a macroscopic CPW prepared on a rigid board connected via an end launch adapter to a microwave signal generator (Anritsu MG3692C). For propagation measurements, the microwave signal was applied in 300-ns long pulses by opening and closing of a switch (SR-T400-1S, rise time ≤4 ns) and the BLS counts were registered as a function of time with 0.8 ns resolution. With the help of phase-resolved BLS measurements (Supplementary Fig. 5), we extracted the exchange constant and the effective magnetization of the thin YIG in sample 1: *J* = (2.7 ± 0.2) × 10^−7^ erg cm^−1^, and *μ*_0_*M*_eff_ = (0.180 ± 0.002) T. The values were consistent with the values reported in the literature^27,34,49^. We used the experimentally determined values for the calculation of wavevectors. For the extraction of the frequency of PSSW1 modes in thin YIG for different effective fields, we measured the thermally excited magnon modes^50^ using spatially resolved BLS. Due to the thermal fluctuations, a spin system possesses a large variety of magnons at a finite temperature. These are spread over a large momentum and energy space, depending on the temperature. We proceeded as follows. We first looked for broad peaks associated to the thermally excited magnon continuum. We attributed the first peak after the continuum to the PSSW1 and extracted manually the peak frequency. Then a local fit with a Gaussian function in a frequency window of 1 GHz was conducted to get a precise value of the peak frequency and its standard deviation. We note that PSSW1 is not resolved in VNA spectroscopy data due to its small spectral weight in inductive measurements.

### Micromagnetic simulations

Micromagnetic simulations were conducted using Mumax^3^ (version 3.9.3)^51^. The nominal cross-section (*y*–*z* plane) of sample 1 (using rectangular geometries for the Fe lines) was discretized into (1, 16,384, 512)(*x*, *y*, *z*) cells with a cell size of 25 × 2.5 × 2.5  nm^3^. Along the *x*-direction, periodic boundary conditions (1024 repetitions) were used, implying the magnetization did not vary along the *x*-direction. The periodic repetitions were added in negative and positive *x*-direction, and considered for the calculation of the demagnetization field^52^ by Mumax3. For YIG *M*_s_ = 143.2 kA m^−1^ and *A*_e*x*_ = 2.7 pJ m^−1^ were used. For Fe *M*_s_ = 1, 710 kA m^−1^ and exchange stiffness *A*_e*x*_ = 21 pJ m^−1^^53^ were considered. Exchange coupling between Fe and YIG was considered in the simulations with the default scaling factor *S* = 1 following ref. ^51^. For each field direction, the magnetic ground state was found using the “Minimize()” method of Mumax^3^. The cell sizes in *y*-  and *z-*directions were smaller than the exchange lengths of Fe (3.4 nm) and YIG (14.5 nm). When using 50 × 5 × 5 nm^3^, the effective field and its spatial dependence were similar; the simulated values in region R_G_ agreed within 0.3%.

### Magnon dispersion relation and group velocity calculations

The magnon dispersion relations of backward volume mode were taken from ref. ^38^:

1 $$\omega =\sqrt{({\omega }_{{\rm{H}}}+\beta {\omega }_{{\rm{M}}}{k}^{2})\left[{\omega }_{{\rm{H}}}+\beta {\omega }_{{\rm{M}}}{k}^{2}+{\omega }_{{\rm{M}}}\left(1-\frac{kd}{2}\right)\right]}$$

In our case, *k* is the in-plane wavevector of magnons along *y*-direction. *ω*_H_ = *γB*_eff_, *ω*_M_ = *μ*_0_*M*_eff_ and $\beta =(2J){({\mu }_{0}{M}_{{\rm{s}}}^{2})}^{-1}$. *μ*_0_ is the permeability of free space. *B*_eff_ is the effective magnetic field in the YIG thin film. *M*_eff_ is the effective magnetization. *J* is the exchange stiffness. *d* is the thickness of the YIG thin film. The group velocity *v*_g_ is calculated from Eq. (1) according to

2 $${v}_{{\rm{g}}}=\frac{\partial \omega }{\partial k}\approx \frac{\Delta \omega }{\Delta k}.$$

Evaluated group velocities are plotted in Fig. 5b as a solid black curve.

## Supplementary information

Supplementary Information

## Data Availability

Requests concerning data should be addressed to D.G. The data sets analyzed in the current study are available in the Zenodo repository, 10.5281/zenodo.3633075. The source data underlying Figs. 1d, 3b, 4c, 5b and 6b and Supplementary Figs. 1 and 5b are provided as a Source Data file.

## Code availability

The code used for micromagnetic simulations is found in ref. ^51^, and input scripts are available in the Zenodo repository, 10.5281/zenodo.3633075.
