# Supplementary material for: Efficient wavelength conversion of exchange magnons below 100 nm by magnetic coplanar waveguides
Source: Nat Commun. 2020 Mar 19;11:1445. doi: 10.1038/s41467-020-15265-1 (PMC7081200; doi:10.1038/s41467-020-15265-1)
Supplement: Supplementary file 1 — Supplementary Information [file 41467_2020_15265_MOESM1_ESM.pdf]

Supplementary Information

Efficient wavelength conversion of exchange magnons below 100 nm by magnetic coplanar waveguides

Ping Che et al.

Supplementary Tab. I: Parameters of samples investigated. In sample3 and sample6 with mCPWs consisting of 17 nm thick Fe, the conversion of magnon wavelengths was smallest as the dipolar fields did not vary the effective fields as much as in case of thick Fe. In their case the frequency separation between branches A and B at 90 deg amounted to only 0.4 GHz (Supplementary Fig. 1).

Device No.	Thickness of YIG film (nm)	Thickness δ of Fe in mCPW (nm)
sample1	100	155
sample2	100	95
sample3	100	17
sample4	100	Non
sample5	36	95
sample6	36	17
sample7	36	non

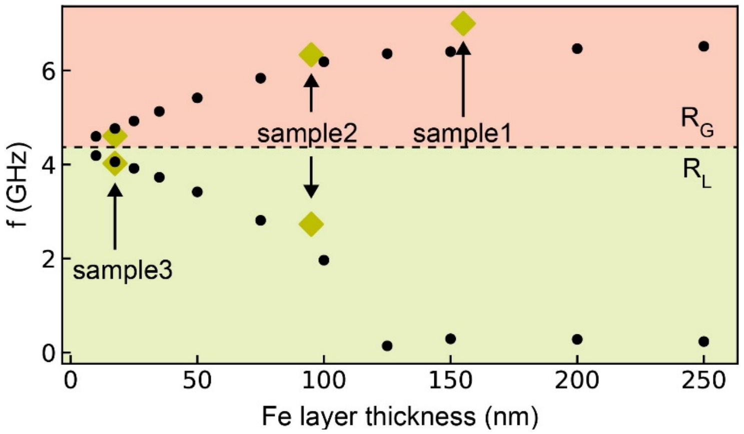

Supplementary FIG. 1. Dependence of high and low frequency branches on Fe layer thickness δ in a 100 nm-thick YIG film. Using the parameters and geometry of Fig. 3**a**, the effective field was simulated for different thicknesses δ of the Fe layer. The field was kept constant at 0.09 T applied under θ = 90 deg. The effective field in the YIG film was extracted in the middle of the regions R_G and R_L (cf. Fig. 3**b**), and the resonance frequency for k = 0 rad μm^{-1} was calculated (black circles). Up to a Fe thickness of about 130 nm the mode splitting is found to increase with the Fe thickness. For larger thicknesses a saturation effect is observed. The yellow diamonds indicate the highest and lowest resonance signal in S_{11} for sample1, sample2 and sample3 at 0.09 T under θ = 90 deg. For sample1 the lowest branch was not resolved at θ = 90 deg (c.f. Fig. 2**a**). Overall there is a good agreement between resonance frequencies calculated from simulated effective fields and and the experimental data (diamonds). Source data are provided as a Source Data file.

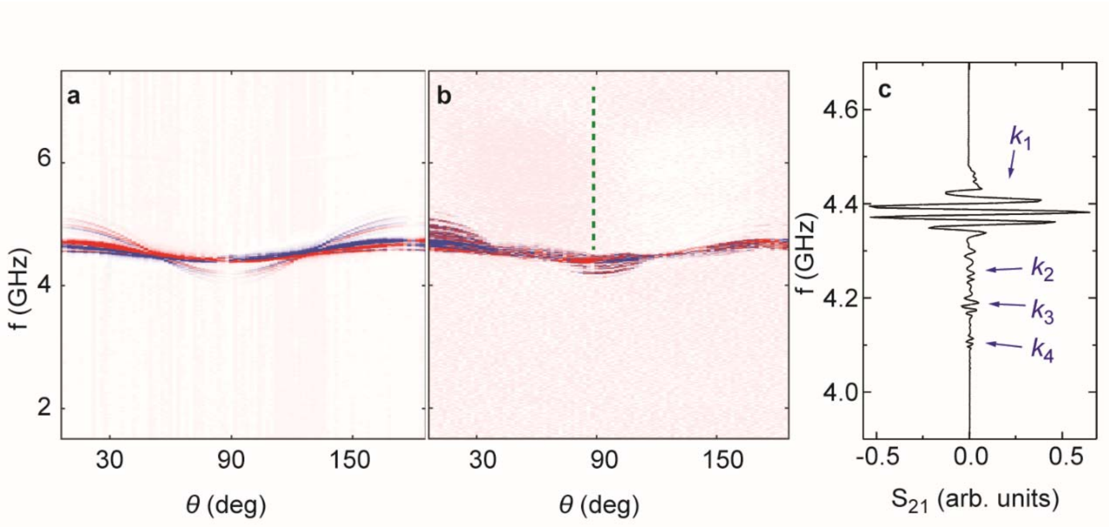

Supplementary FIG. 2. Spectra of magnons emitted and detected by conventional non-magnetic CPWs (sample4). The CPWs consist of Ti(5nm)/Au(120nm). **a** Angular-dependent reflection signals ΔS_{22} and **b** transmission signal ΔS_{21} at $\mu_0 H$ = 0.09 T measured on 100 nm-thick YIG with a non-magnetic CPW. Red (blue) color represents 1 (-1). The dashed vertical line indicates where the bottom line spectrum of Fig. 1**b** is extracted. **c** Detailed spectrum of S_{21} at $\mu_0 H$ = 0.09 T measured with the non-magnetic CPWs of sample4. The strongest branch belongs to the excitaton at k_1. The weaker ones to k_2, k_3 and k_4, i.e., wavevectors transferred by a non-magnetic CPW.

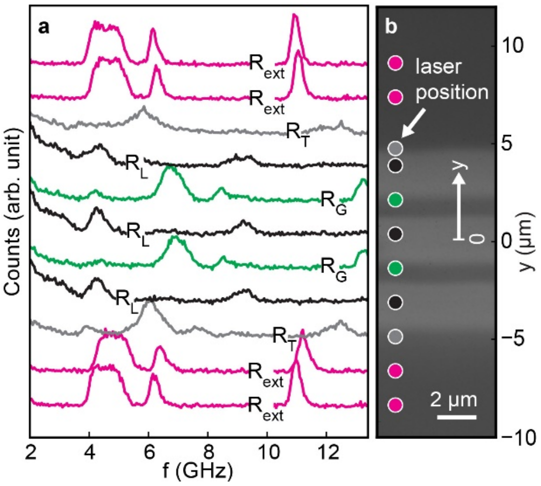

Supplementary FIG. 3. Spatially resolved magnon spectra at the mCPWs at 0.087 T. a Spectra of thermally excited magnons in sample1 measured by μBLS for μ_0H = 0.087 T and θ = 90 deg at different laser spot positions indicated by colored circles in **b** the optical image taken by the BLS microscope. The data shows similar characteristics as discussed for μ_0H = 0.15 T in the main text. We consider the same color-code concerning positions and spectra as in the main text. The lowest frequency mode in region R_L was not fully resolved due to the frequency range of the interferometer setting, restricted to f > 2 GHz.

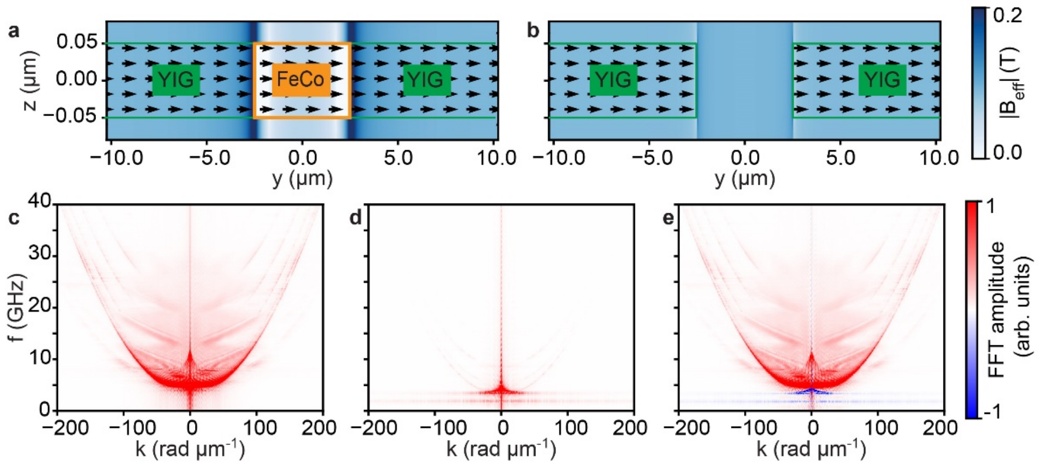

Supplementary FIG. 4. Micromagnetic simulation of spin wave emission by an embedded FeCo stripe.

a Cross-sectional view of a 100 nm thick YIG film with a 5 µm wide FeCo stripe with $\mu_0 M_S$ = 2.45 T fully embedded. Arrows are extracted from micromagnetic simulations and display locally averaged magnetization components in the *y*-*z*-plane. A field of 0.09 T is applied in positive *y*-direction (θ = 90 deg). The color-coding of the background (legend on the right) indicates the magnetic field $|B_{eff}|$. **b** For comparison, the same YIG film is simulated with an air gap of 5 µm instead of the FeCo. Using the static magnetization configurations of panels **a** and **b**, we conducted dynamic simulations for both cases by applying a Sinc-pulse [1] with a cut-off frequency of 40 GHz. The dynamic field was applied uniformly under 45 degree in the *y*-*z*-plane with a peak amplitude of 1 mT. The simulations were conducted for 6 ns and the normalized magnetization vectors **m** stored every 5 ps for each cell. We performed a Fourier transform of the magnetization component m_z in time and *y*-direction. The Fourier amplitudes are displayed in **c** for YIG with embedded FeCo and in **d** for YIG with an air gap. In **e** we show the difference between the data displayed in **c** and **d**, highlighting the effect of the FeCo. All spectra of **c** to **e** are plotted with the same colorscale. From **e** we extract that the FeCo stripe induces additional large-wavevector spin waves in YIG compared to the air gap. Excitation at wave vectors up to 170 rad μm^{-1} is visible in **e**. The corresponding wavelength amounts to 37 nm in YIG.

Simulation details:

For FeCo we assumed M_s = 1950 kA m^{-1}, A_{ex} = 21 pJ m^{-1} and Gilbert damping parameter α = 0.01, for YIG we used M_s = 143.2 kA m^{-1}, A_{ex} = 2.7 pJ m^{-1} and α = 0.0005. The sample was discretized into (1; 8192; 32)(x; y; z) cells with a mesh size of 25 x 5 x 5 nm^3. In *x*-direction a periodic boundary conditons was used (1024 repetitions into into both side). At the outer boundaries in *y*-direction the Gilbert damping of the YIG was gradually increased to absorb spin waves and avoid back-reflection following [2].

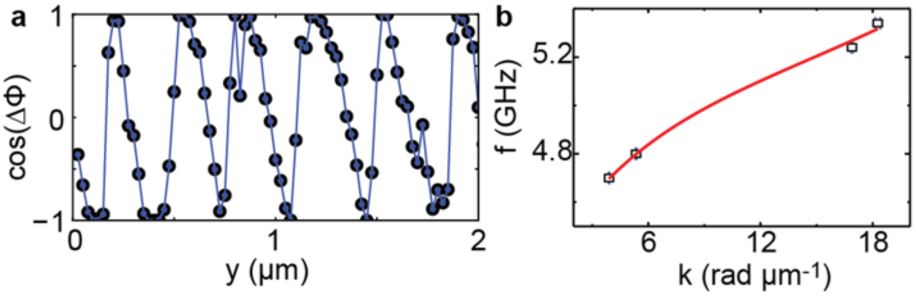

Supplementary FIG. 5. Phase evolution of magnons emitted by a non-magnetic CPW.
a Phase-resolved BLS data measured in region R_{ext} at an excitation frequency of 5.34 GHz and μ_0H = 0.087 T. The spatial variation of the magnon phase is consistent with a wavelength of about 340 nm, i.e., a wavevector of 18.3 (± 0.14) rad μm^{-1}. **b** Symbols show measured wavevectors of magnons excited at different frequencies in the phase-resolved BLS. The wave vectors were extracted by linear fitting of the unwrapped phase $\Delta\varphi$(y), the error bars in *k* indicate the standard deviation of the fitting. The solid line reflects the dispersion relation consistent with the Damon-Eshbach geometry (θ = 0 deg) following the formalism of Kalinikos and Slavin [3]. Source data are provided as a Source Data file.

References:

[1] Venkat, G. *et al.* Proposal for a Standard Micromagnetic Problem: Spin Wave Dispersion in a Magnonic Waveguide. *IEEE Trans. Magn.* **49**, 524 (2013).

[2] Venkat, G., Fangohr, H., & Prabhakar, A. Absorbing boundary layers for spin wave micromagnetics. *J. Magn. Magn. Mater.* **450**, 34-39 (2018).

[3] Kalinikos, B. A. & Slavin, A. N. Theory of dipole-exchange spin wave spectrum for ferromagnetic films with mixed exchange boundary conditions. *J. Phys. C: Solid State Phys.* **19**, 70137033 (1986).
